# Supplementary figures and images for: Response Surface Methodology for Optimization of Process Parameters and Antioxidant Properties of Mulberry (Morus alba L.) Leaves by Extrusion
Source: Molecules. 2020 Nov 10;25(22):5231. doi: 10.3390/molecules25225231 (PMC7697072; doi:10.3390/molecules25225231)

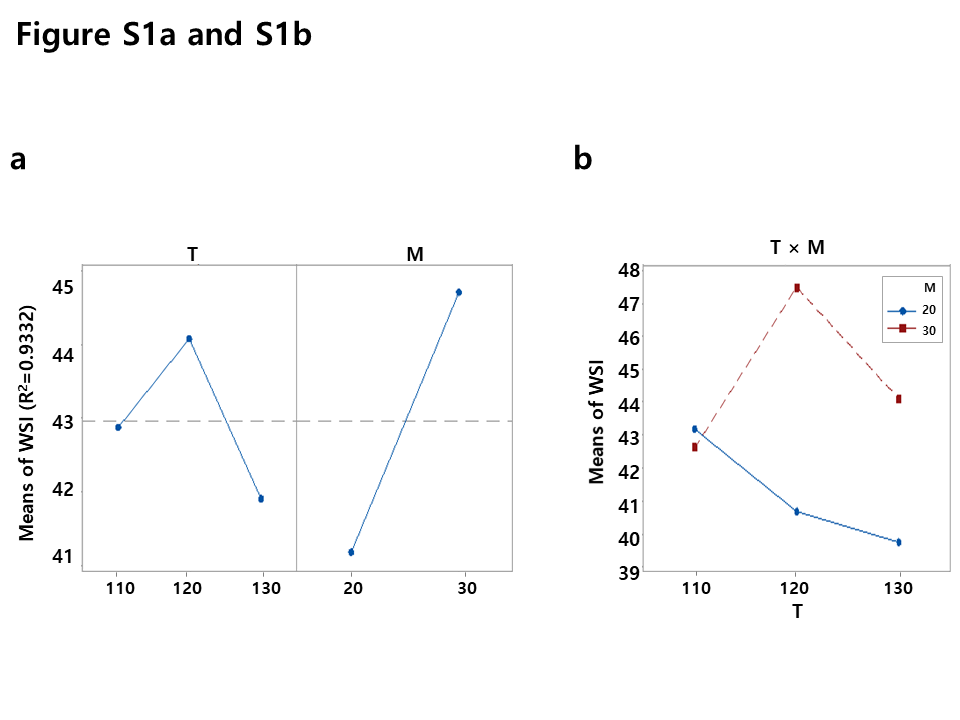

Supplement: Supplementary file 1 [file molecules-25-05231-s001.zip › supplements - Mina Kim (molecules) -revised/supple Figure S1a and S1b (Mina Kim).TIF]

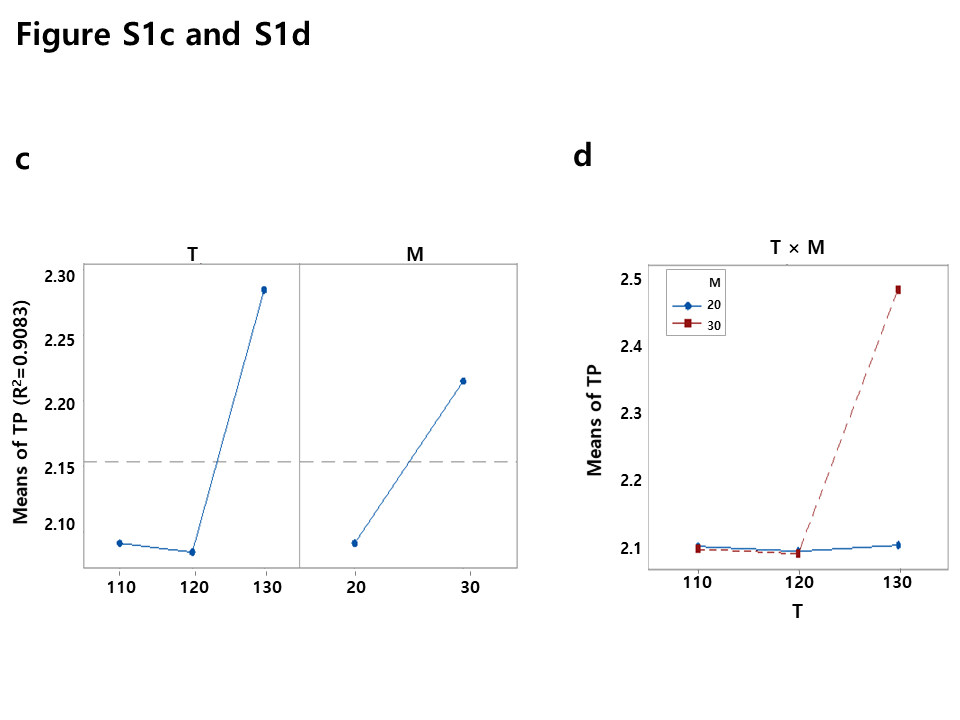

Supplement: Supplementary file 1 [file molecules-25-05231-s001.zip › supplements - Mina Kim (molecules) -revised/supple Figure S1c and S1d (Mina Kim).TIF]

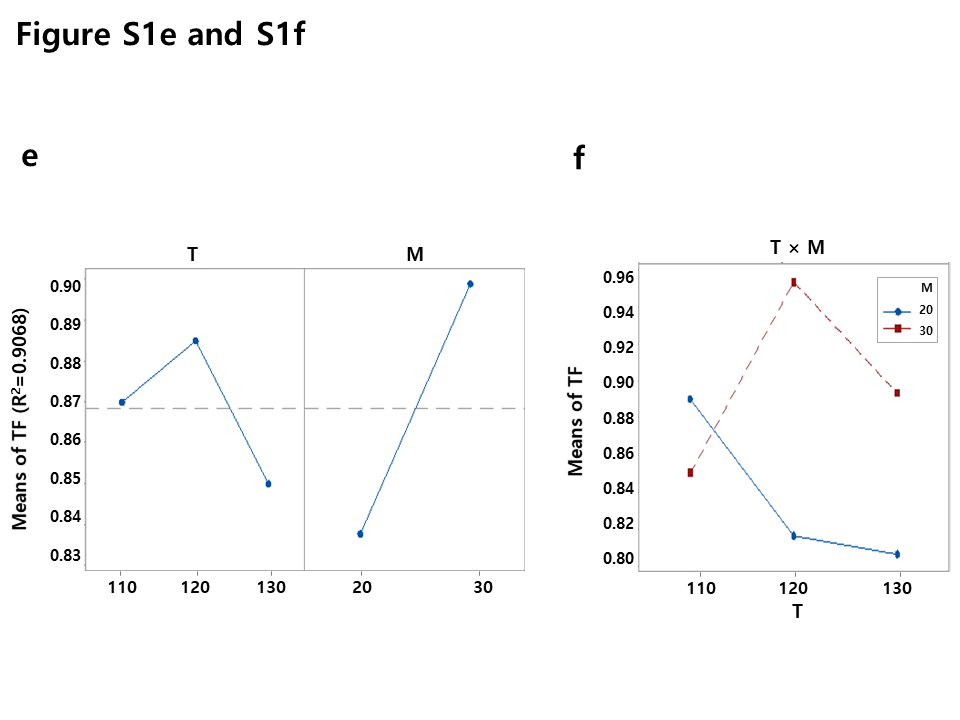

Supplement: Supplementary file 1 [file molecules-25-05231-s001.zip › supplements - Mina Kim (molecules) -revised/supple Figure S1e and S1f (Mina Kim).TIF]

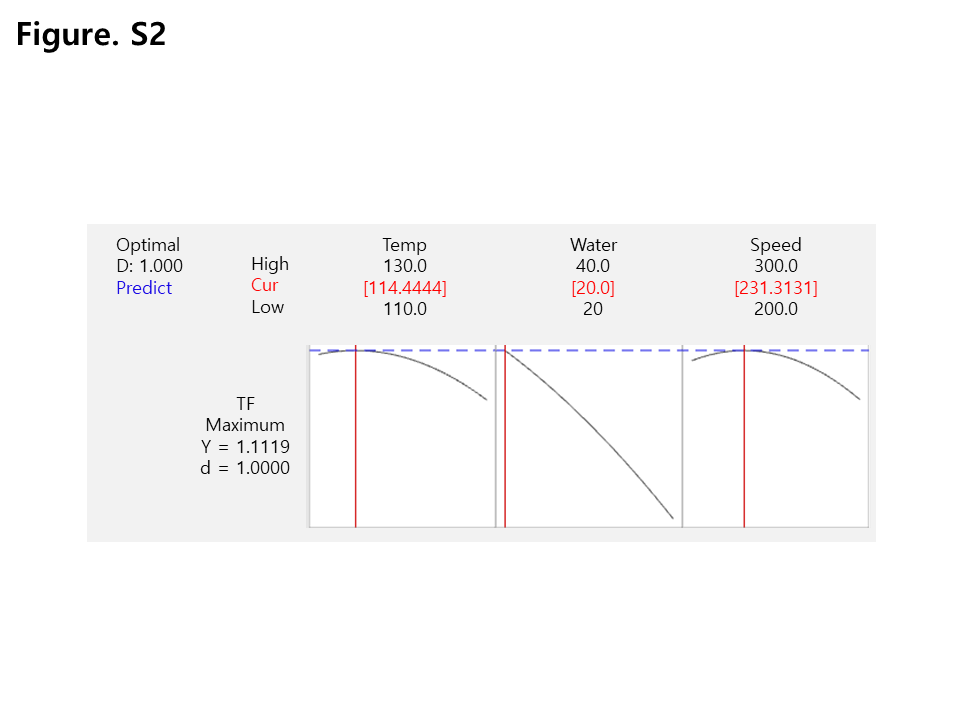

Supplement: Supplementary file 1 [file molecules-25-05231-s001.zip › supplements - Mina Kim (molecules) -revised/supple Figure S2 (Mina Kim).TIF]

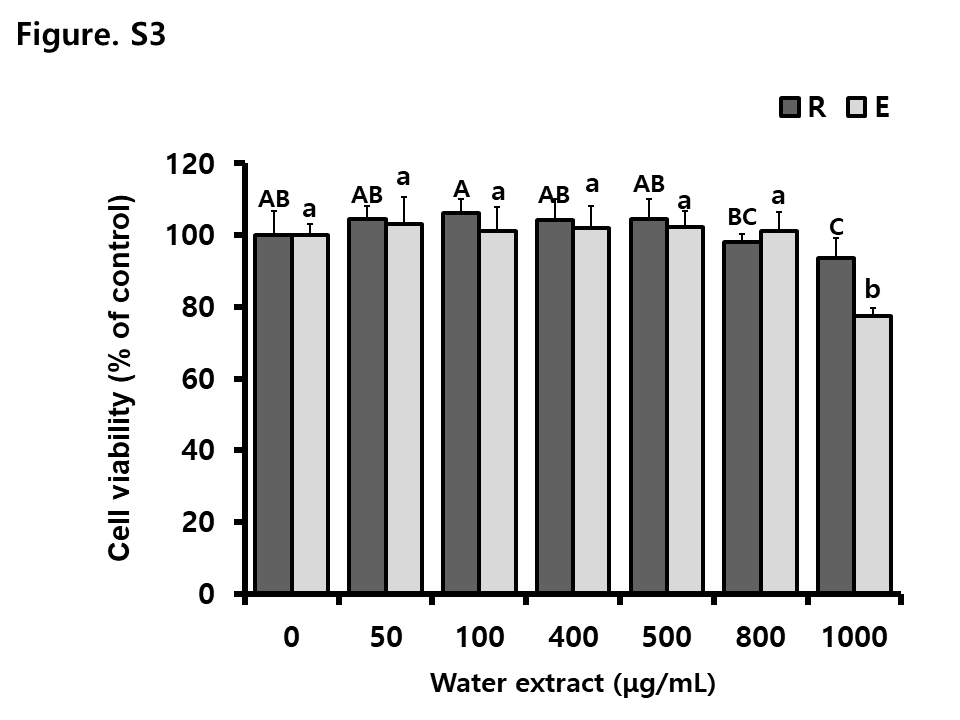

Supplement: Supplementary file 1 [file molecules-25-05231-s001.zip › supplements - Mina Kim (molecules) -revised/supple Figure S3 (Mina Kim).TIF]

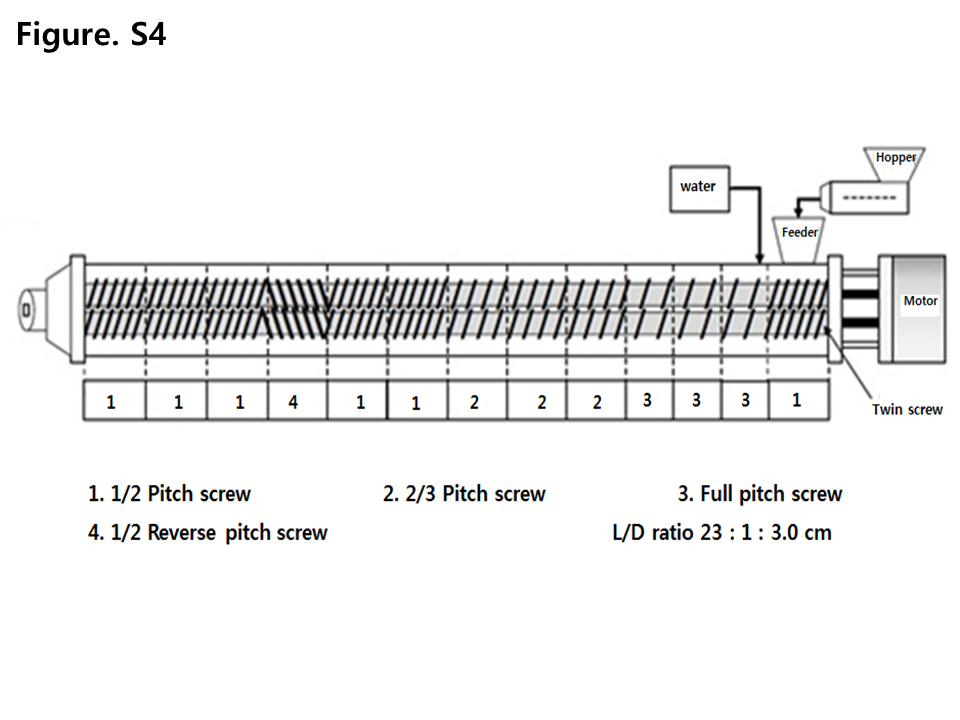

Supplement: Supplementary file 1 [file molecules-25-05231-s001.zip › supplements - Mina Kim (molecules) -revised/supple Figure S4 (Mina Kim).TIF]
